# Spatial Differentiation of Microbial Communities in Hybrid Membrane Bioreactor (HMBR) and Their Impact on Pollutant Removal

**DOI:** 10.3390/membranes16020068

**Published:** 2026-02-19

**Authors:** Ying Li, Yuhan Liu, Qiang Liu, Wei Xiang, Jixiang Qu, Yangyang Yang, Xiulei Fan, Huixian Li, Hongmei Du

**Affiliations:** 1 School of Environmental Engineering, Xuzhou University of Technology, Xuzhou 221018, China; 11469@xzit.edu.cn (Y.L.);; 2 Professional Education and Field Training Base, Army Engineering University of PLA, Xuzhou 221000, China; 3 Jiangsu Diqing Environmental Technology Co., Ltd., Xuzhou 221000, China; 4 Xuzhou River and Lake Management Center, Xuzhou 221018, China; 5 Shandong Jianzhu University Design Group Co., Ltd., Jinan 250101, China

**Keywords:** microbial community structure, mathematical analysis, functional bacteria, hybrid membrane bioreactor, high-throughput sequencing

## Abstract

A hybrid membrane bioreactor (HMBR) enhances treatment performance by simultaneously utilizing organisms on both suspended and attached sludge, yet the microbial mechanisms underpinning their efficiency remain poorly understood. In this study, we investigate spatial variability within microbial communities in HMBRs and correlate this factor with pollutant removal capacity. High-throughput sequencing results revealed significant differences in community structure between suspended sludge, suspended media surfaces, and membrane module surfaces. Suspended sludge exhibited the highest species richness, whereas microbial communities on suspended media resembled those within the sludge, contrasting markedly with membrane surface communities. Key functional groups were enriched at specific locations: *Pseudomonas* and *Comamonas* dominate the surface of the suspension culture medium and participate in nitrification; phosphorus-accumulating organisms (PAOs), primarily from the *Flavobacteriales* and *Planctomycetaceae* phyla, were most abundant on suspended media surfaces. This spatial partitioning of functional microbes indicates cooperative division of labor. Media surfaces serve as primary sites for nitrification and phosphorus removal, whilst suspended sludge flocs and membrane module surfaces are the principal contributors to denitrification. The results of this study provide microbiological evidence for optimizing HMBR design and operation, confirming that spatial community structure is a key factor influencing performance.

## 1. Introduction

MBR is an abbreviation for membrane bioreactor, which combines a membrane separation process with activated sludge to achieve better pollutant removal efficiency [1]. It can greatly reduce sludge production and is widely used in urban sewage treatment and industrial wastewater treatment [2]. The improvement and optimization of MBR technology is a current research area [3], with considerable research focused on enhancing treatment efficiency and controlling membrane fouling [4,5]. Among the proposed solutions, the introduction of suspended biofilm carriers into the MBR system has proven effective [1,5]. In one study, following a long operating period, large numbers of microorganisms survived and reproduced in such a reactor, including bacteria, protozoa, and a small number of metazoans. These microorganisms are not only scattered in the mixed liquor but also attached to the surfaces of suspended fillers and membrane modules. Therefore, both attached and suspended activated sludge were present in the bioreactor. This hybrid membrane bioreactor is also abbreviated to HMBR. The HMBR has gained increasing attention for advanced wastewater treatment, particularly where high effluent quality, compact design, or process intensification is required [6]. Its applications are continually expanding into industrial and municipal sectors, particularly those subject to stringent emission regulations. Compared to a conventional MBR, suspended media consistently provide key operational benefits: enhanced process stability and shock load resilience, reduced membrane fouling, and effective treatment at higher organic loading rates [7,8].

In MBR and HMBR systems, pollutant removal relies not only on the interception function of membrane modules but is also closely linked to the metabolic activities of various functional microorganisms within suspended and attached activated sludge [9]. The microbial structure within activated sludge directly influences pollutant degradation pathways and efficiency [10]. Preliminary research results [6,11] have shown the optimal operating conditions of HMBR and MBR systems within the field of pollutant removal efficiency and membrane fouling control capabilities. The optimal SRT of an HMBR is 30 days; in comparison, that of an MBR is 10 days. However, the results of comparative studies of the pollutant removal efficiencies of an MBR and HMBR under optimal operating conditions from a community structure perspective remain scarce. With the progressive refinement of high-throughput sequencing technologies, microbial community structure analysis has become more accurate and efficient, providing robust technical support for elucidating microbial distribution and function within systems [12]. The division of mechanisms regarding carbon removal, denitrification, and phosphorus removal among different units, such as the membrane surface, suspended media surface, and mixed liquor sludge surface within HMBR, is particularly unclear.

Previous research on suspended media in MBR has primarily focused on macro-scale performance improvements, such as reduced extracellular polymeric substances (EPS) production, enhanced sludge filterability, extended filtration cycles, improved low-temperature nitrification, and better removal of recalcitrant pollutants [13,14]. However, these studies largely correlate media presence with system outcomes without elucidating the underlying microbiological mechanisms. There remains insufficient understanding of the mechanisms by which media function, specifically whether they merely increase biomass or optimize microbial ecosystems through functional spatial partitioning. This knowledge gap constrains the refinement process for HMBR.

In this study, microbial community structures are compared across three functional units within the HMBR system—membrane module surfaces, suspended packing surfaces, and mixed liquor sludge surfaces—to explore the variance among them. The mechanisms of efficient carbon reduction, nitrogen removal, and phosphorus removal within HMBR and MBR systems are specifically discussed from the perspective of functional microorganisms. Furthermore, the functional division and synergistic interactions in carbon, nitrogen, and phosphorus removal pathways are elucidated, thereby providing a biological rationale for the enhanced performance mechanisms of HMBR.

## 2. Materials and Methods

### 2.1. Experimental Apparatus and Operating Conditions

**Figure 1:**
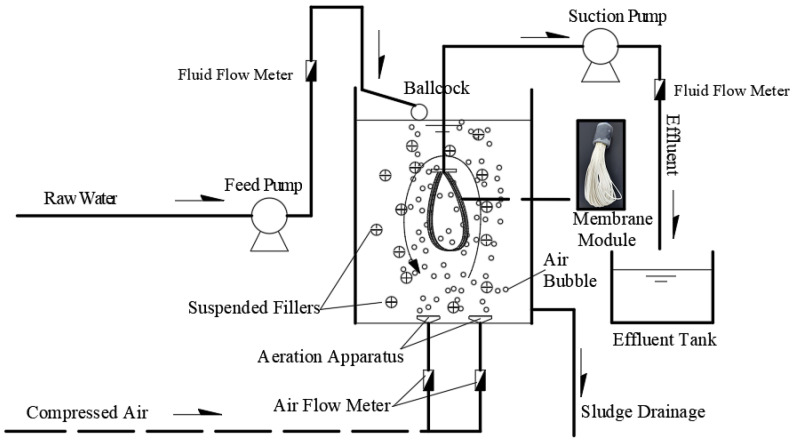
Schematic diagram of the HMBR process. Commentary: The arrows mean the direction of the water and the compressed air.

The schematic diagram of the HMBR process employed in this study is shown in Figure 1. The structure of the MBR is identical to that of the HMBR, excluding the inclusion of suspended fillers. Raw water was pumped into the reactor by the feed pump and then mixed with compressed air from the bottom of the HMBR and transferred through the membrane module under the pressure of the suction pump. The membrane module consists of polyvinylidene fluoride (PVDF) hollow fiber microfiltration membranes with a diameter of 4.0 mm, a pore size of 0.2 μm, and a membrane surface area of 1.0 m^2^. Driven by a peristaltic suction pump, it performs solid–liquid separation in the mixed solution, with an operation cycle of 5 min (4 min on; 1 min off). The transmembrane pressure (TMP) is monitored in real-time via a vacuum pressure gauge. When the TMP reaches 20 kPa the fouled membrane module is replaced by a new one, and the subsequent operation cycle begins.

The operating parameters of the HMBR in this study are presented in Table 1. The raw water came from a university campus and was filtered by a 1 mm sieve to remove the particles. Its characteristics are listed in Table 2. The inoculum sludge was sourced from a local municipal sewage treatment plant.

### 2.2. Detection Methods

The pH value and temperature were measured using the Sima HP818 portable pH pen. Dissolved oxygen was measured using a portable dissolved oxygen analyzer (HQ30d). NH_4_^+^-N was determined by means of Nessler’s reagent spectrophotometry, using ultraviolet visible spectrophotometer UV-1900i produced by Shimadzu Co., Ltd., Jingdu, Japan. TP was analyzed using the molybdenum antimony spectrophotometric method. COD_Cr_ and TN were measured through Lianhua Technology 5B-6C three-parameter water quality analyses produced by Lianhua Technology Co., Ltd., Beijing, China. The frequency of these routine indicator measurements of effluent from the effluent tank is once per day.

Activated sludge samples were collected from the residual sludge discharge port at the bottom of the reactor at the end of the operating cycle. Microscopic photographs were taken using an optical microscope produced by Beijing Padiwei Instrument Co., Ltd., Beijing, China. The sludge samples adhered to the surface of the suspended fillers, and the membranes were carefully scraped and enriched with a plastic blade. All sludge samples were stored in a sterile centrifuge tube at −20 °C. The sludge samples collected from the membrane surface, suspended fillers’ surface, and activated sludge mixture in the HMBR under an SRT of 30 days were marked as C1, C2, and C3, respectively. The sludge samples collected from the membrane surface and activated sludge mixture in the MBR under an SRT of 10 days were marked as D1 and D2. All samples were sent to Tiny Gene Bio-Tech (Shanghai) Co., Ltd. (Shanghai, China) on dry ice for DNA extraction and high-throughput sequencing and related data analysis as required.

DNA was extracted using the Proteinase K cleavage method, and 3 μL was detected using 1.2% agarose gel electrophoresis; thereafter, two-way sequencing was carried out for the target area and the fusion material with the primer pair 515F 5′-GTGCCAGCMGCCGCGGTAA-3′ and 926R 5′-CCGTCAATTCMTTTGAGTTT-3′ targeting the V4–V5 region of bacterial 16S rRNA genes. The PCR cycle details are listed in Table 3, and PCR amplification conditions are shown in Table 4. The other test processes and conditions are identical to those in reference [15].

### 2.3. Data Analysis Methods

Operational taxonomic units, abbreviated to OTUs, are used for convenient and straightforward analysis and discussion in phylogenetics or population genetics research. OTUs represent groupings of sequences assigned to a taxonomic unit, such as strain, genus, species, etc., based on different levels of similarity, typically at 97%. Selected from the Silva database, the OTUs were obtained randomly from the sequences; thereafter, the species accumulation curve was drawn by using the software R (version: 3.6.0) and the language vegan (version: 2.5-5). When the species accumulation curve continues to rise sharply, in an almost straight manner, this indicates that the sampling size is insufficient and must be increased. If the curve changes from a sharp rise to a gradual rise, this indicates that sampling is sufficient. Therefore, the sequencing depth of the samples can be determined by reading the species accumulation curve. Selecting an OTU sample with a similarity level of 97%, using software R (version: 3.6.0) language, VENN (version: 1.7), and plotrix (version: 3.7-5) packages to draw Venn diagrams, can effectively denote the similarity and overlap of OTU numbers. Microbial diversity is commonly studied in community ecology, and the abundance and diversity of microbial communities can be reflected through a single sample diversity analysis referred to as alpha diversity. This index includes a series of statistical analysis indices, such as the Chao index, ace index, Shannon index, Simpson index, PD-whole-tree index, etc. Beta diversity analysis is also used to compare the magnitude of differences in species diversity between different samples. Hierarchical clustering analysis based on community composition (Bray–Curtis algorithm) and the community structure bar chart of the samples were plotted in one graph. Bray–Curtis distance is a commonly used indicator to reflect the differences between different communities. The calculation of Bray–Curtis distance does not take into account the evolutionary distance between sequences, only the presence of species in the sample. The value of Bray–Curtis distance is between 0 and 1, with larger values indicating greater differences between samples. Levels of phylum, class, and order analysis results were discussed in this study. Raw sequencing data have been uploaded to the NCBI database, and the corresponding project accession number is SRP662027: PRJNA1402667.

## 3. Results and Discussion

### 3.1. Removal Efficiency of Contaminants

In this study, we compared the operational performance of an HMBR and MBR under identical sludge retention time (SRT = 10 days) and hydraulic retention time (HRT = 10 h) conditions. As shown in Table 5, during the stable operation phase, the HMBR system demonstrated superior average effluent parameters and removal efficiencies across all indicators compared to the MBR system. This advantage was particularly pronounced in the degradation of NH_4_^+^-N, achieving an average removal rate of 98.9%. Although the removal efficiencies for TN and TP were lower than that of NH_4_^+^-N, they remained higher than those of the MBR system.

**Figure 2:**
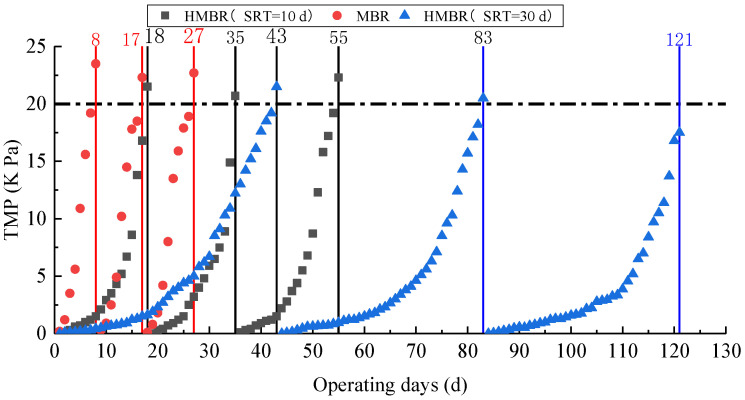
Comparison of TMP between the MBR and HMBR.

The TMP of the HMBR and MBR can reflect the membrane fouling characteristics. Three operating cycles of the HMBR and MBR are presented in Figure 2. As shown in Figure 2, the TMP rise rate of the HMBR is markedly slower under the SRT of 10 days, with an operational cycle approximately 2.7 times that of the MBR. This finding indicates that the addition of suspended fillers effectively retards the membrane fouling process. Under an SRT of 30 days, the membrane fouling control advantage of the HMBR becomes even more pronounced, almost fifteen times longer than that of the MBR.

The combined results indicate that the HMBR system demonstrates superior pollutant removal efficiency and membrane fouling control compared to the MBR system. This finding may be attributed to the addition of suspended fillers, which enables the coexistence of both suspended and attached microorganisms within the HMBR system. This factor not only increases the biomass within the system but also enriches the microbial community structure and the ecological niche distribution of functional microbial populations [15,16]. Consequently, this process enhances the degradation capacity of organic matter, improves the characteristics of the mixed liquor, and delays membrane fouling.

### 3.2. Microbial Structure Analysis

#### 3.2.1. Microbial Analysis

**Figure 3:**
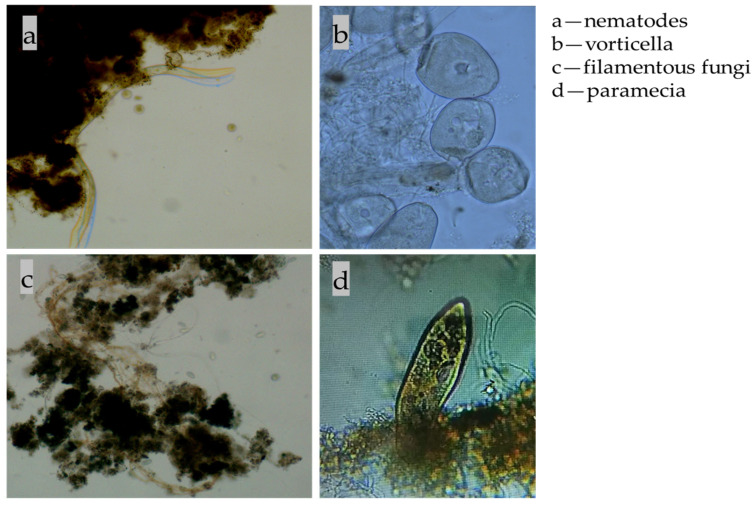
Microscopic images of selected microorganisms in the reactor.

Through microscopic examination of the HMBR system, various microorganisms were identified, as shown in Figure 3, including nematodes, vorticella, filamentous fungi, and paramecia. The stable presence of these organisms indicates, to a certain extent, that the ecological environment within the system is favorable and water quality is improved and tending towards stability.

From an ecological perspective, these microorganisms each play vital roles within the system. When native and post-protozoa are found in the activated sludge system, this indicates that the food chain of the system is more complete, and the effluent effect is better and more stable. Nematodes promote organic matter decomposition and nutrient cycling by grazing on microorganisms. As a foundational link in the food chain, they contribute to maintaining ecological equilibrium. *Vorticella*, a common sessile ciliate in activated sludge, enhances sludge flocculation while extensively preying on free-living bacteria, thereby improving effluent quality [17]. It serves as an excellent indicator organism for wastewater treatment efficacy. *Filamentous bacteria*, as a vital component of the activated sludge microbial community, contribute to maintaining the stability of floc structure, thereby safeguarding biochemical treatment efficiency [18]. *Paramecium*, feeding on bacteria and algae with a strong phagocytic capacity, exerts a direct purifying effect on water bodies [19].

The coexistence and activity of these microorganisms indicate that the HMBR system has established a complex and stable microfood chain, enhancing not only overall pollutant removal efficiency but also strengthening the system’s buffering capacity against water quality fluctuations. Consequently, at the microbial ecological level, this finding underpins the superior treatment performance of the HMBR compared to the conventional MBR.

#### 3.2.2. Species Accumulation Curve Discussion

**Figure 4:**
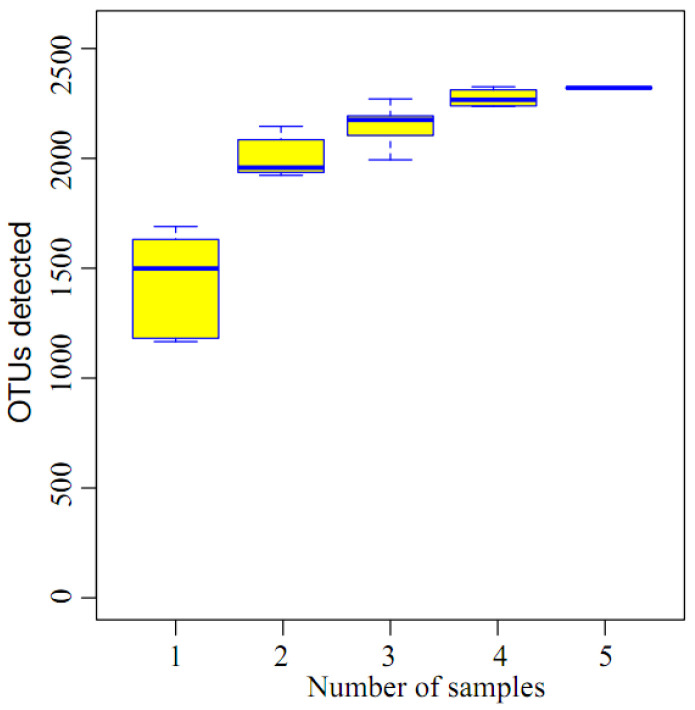
Species accumulation curves of groups C1, C2, C3, D1, and D2.

The species accumulation curves of samples C1, C2, C3, D1, and D2 are presented in Figure 4. The data presented in Figure 4 show that the curve transitions from a sharp rise to a gentle ascent after the steep increase, indicating that almost all species in the samples are already detected, and the sample sequencing quantities are sufficient.

#### 3.2.3. Venn Analysis

**Figure 5:**
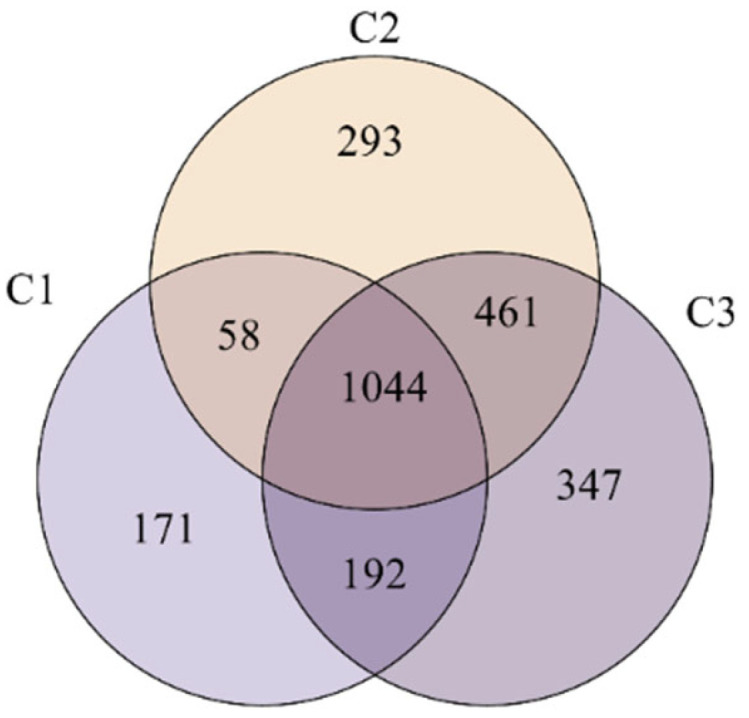
Venn diagram of groups C1, C2, and C3.

Analyzing the Venn graph, the number of identical and sole OTUs in multiple samples can be determined; therefore, the similarity and overlap of OTU composition in the tested samples can be visually represented. Different colors represent different samples (or groups). A Venn diagram of groups C1, C2, and C3 is shown in Figure 5.

By analyzing Figure 5, it can be seen that 1044 identical OTUs are present in membrane surface suspended fillers and activated sludge. The number of sole OTUs from samples C1, C2, and C3 are 171, 293, and 347, respectively. Among the three samples, the most abundant microbial species, with a total of 2044 OTUs, are present in sample C3, whereas the least abundant are detected in sample C1, with a total of 1465. C2 and C3 contain the highest number of similar OTUs between the two samples, standing at 461; in comparison, C1 and C2 contain the lowest number of OTUs at 58. The microbial populations in suspended fillers and activated sludge are more similar, with the lowest abundance of microbial species on the membrane surface and the least similarity to other microbial species in other locations. From the above data, it can be inferred that there are differences in the internal environmental conditions of suspended and attached activated sludge in the HMBR, leading to significant differences in the types and quantities of microorganisms at different locations. Further analysis should be performed to determine differences in diversity and the species responsible for said differences.

#### 3.2.4. Analysis of Alpha Diversity Differences Between Groups

To enable a more effective comparison and analysis of the differences in microbial community structure diversity among groups C1, C2, and C3, the coverage, OTU number at a similarity level of 97%, Chao index, ace index, Shannon index, Simpson index, and PD-whole-tree of the samples are listed in Table 6.

The Chao and ace indices represent the number of species in the community without considering species abundance. The Chao index can only represent the quantities of the species of samples but does not consider the abundance of each species in the community. The Shannon index can reflect both the diversity and evenness of the community. Therefore, under the same species richness, the varieties of the community are positively correlated with the evenness of each species. Among the three samples, the Shannon diversity of the samples from the surface of the suspended carrier is remarkably higher than that of the other two samples, and the number of different species is more evenly distributed. Therefore, it can be inferred that various environments are present in suspended fillers, which can create necessary conditions for the growth and enrichment of various forms of microorganisms with multiple metabolic forms. The Shannon diversity index in the activated sludge mixture is slightly lower, and the ability to adapt to the external environment is slightly weaker. The richness and evenness of species in the sample can both affect the Shannon index and the Simpson index. Under the same species richness, the greater the evenness of each species, the greater the diversity of the community. The PD-whole-tree index is used to calculate community diversity, with a high value indicating higher community diversity.

The markedly higher Shannon diversity observed on C2 indicates that this environment provides a broader range of microhabitats, potentially attributable to gradient distributions of substrate and oxygen concentration within the biofilm. This elevated diversity may contribute to the functional stability and stress resistance of the HMBR system, as communities with greater diversity can maintain metabolic activity under fluctuating conditions.

**Figure 6:**
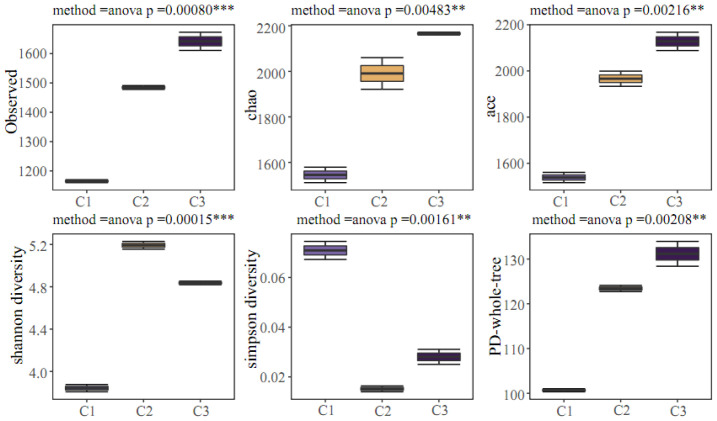
Alpha diversity box diagram of groups C1, C2, and C3. Commentary: *** represent 0 < *p* < 0.001, and ** represent 0.001 < *p* < 0.01.

As can be seen in Figure 6, there is a significant difference in the alpha diversity parameters of C1, C2, and C3, with a *p*-value below 0.05. The *p*-value of the Shannon index is the lowest, with a value of 0.00015.

Based on the comprehensive analysis of the above data, the following conclusion can be drawn: There are differences in the indices representing alpha diversity within each group, and intragroup analysis and comparison are feasible. An evident diversity difference is present in the microbial population neutralized on the membrane surface, suspended fillers’ surface, and activated sludge mixture caused by the various environments of the HMBR. Further clarification is required to determine the species that induce the difference in the microbial community structure at each attachment position of the HMBR.

#### 3.2.5. Beta Diversity Based on Species Information

**Figure 7:**
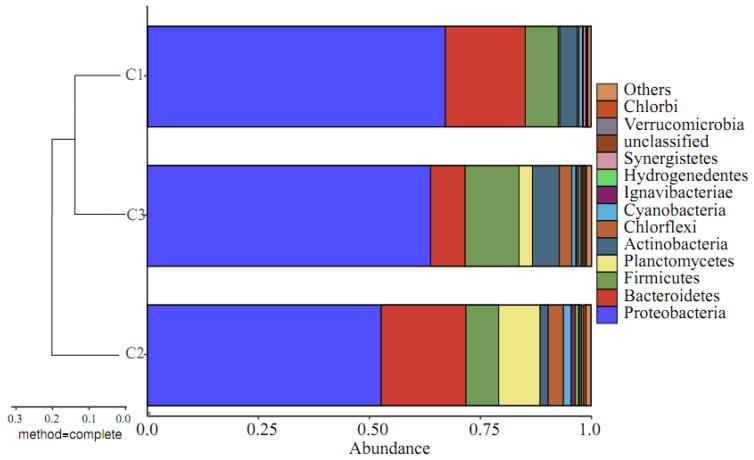
Beta diversity analysis of groups C1, C2, and C3 at the level of phylum.

**Figure 8:**
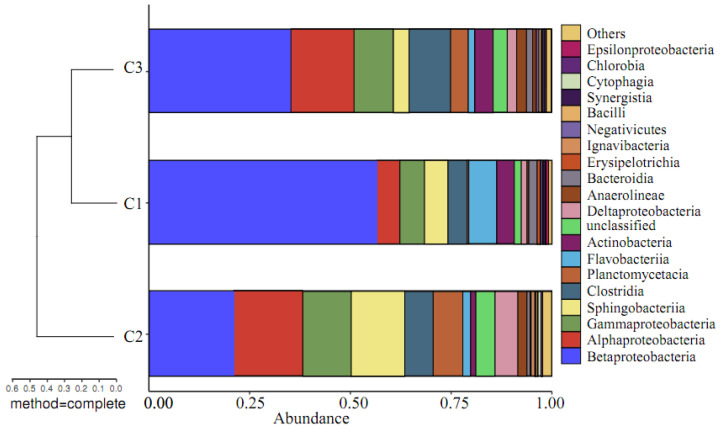
Beta diversity analysis of groups C1, C2, and C3 at the level of class.

**Figure 9:**
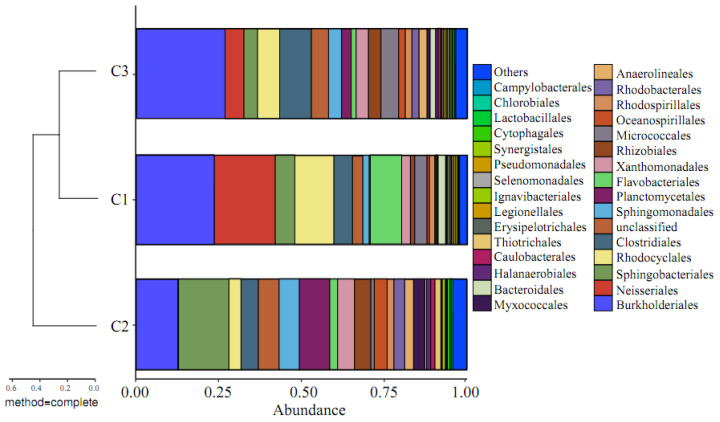
Beta diversity analysis of groups C1, C2, and C3 at the level of order.

Based on the OTU analysis results, it can be seen that, under the same SRT, the types of microbial OTUs in activated sludge at different locations within the HMBR are not identical. To clarify the differences between the three communities, a comparative analysis was conducted on the beta diversity based on species information within the group. Beta diversity analysis of groups C1, C2, and C3 at the level of the phylum is shown in Figure 7, Figure 8 and Figure 9.

As shown in Figure 7, the species causing significant differences at the phylum level are mainly Proteobacteria and Bacteroidetes. At the order level, Burkholderiales, Neisseriales, Sphingobacteriales, Rhodocyclales, and Clostridiales are not only the dominant strains but also the main strains causing significant differences. From the beta diversity analysis of the groups, it can be concluded that microbial communities of membranes and activated sludges were incredibly similar, with a Bray–Curtis value of roughly 0.15–0.25.

#### 3.2.6. Functional Microbial Differences Analysis

**Figure 10:**
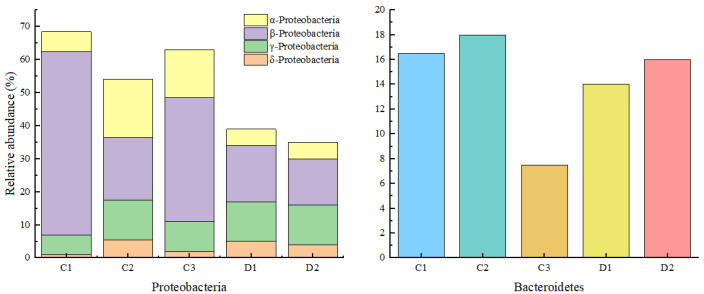
Diagram of the species composition of the differential carbon reduction metabolic pathways in the HMBR and MBR.

The spatial differentiation of microbial communities within the HMBR system generates synergistic multi-stage treatment cascade effects, directly accounting for its exceptional pollutant removal metrics. As shown in Table 5, the HMBR achieves COD_Cr_ removal rates of 94.6–96.1%, significantly surpassing the MBR’s 87.5%. This enhancement exhibits a quantitative correlation with the spatial distribution of carbon-reducing bacteria. Proteobacteria and Bacteroidetes are the primary degraders of COD_Cr_ [20]. As significant differences were found in the removal rate of COD_Cr_*,* it is necessary to investigate the functional distribution differences between these two bacterial phyla within the HMBR system and MBR, along with their potential causes, through differential metabolic pathway species composition analysis. A comparative analysis was conducted to investigate microorganisms with carbon-reducing functions, such as on the membrane surface, suspended filler surface, and activated sludge mixture of the HMBR, and on the membrane surface and activated sludge mixture of the MBR, to more effectively understand the distribution characteristics of carbon-reducing bacteria. The species composition diagram of differential metabolic pathways is shown in Figure 10. Proteobacteria are the absolute dominant bacteria in sludge samples from the membrane surface, suspended filler surface, and activated sludge mixtures. From the analysis results presented in Figure 10. From Figure 10, it can be concluded that Proteobacteria play dominant roles in degrading COD_Cr_ in this HMBR process, especially those that exist on the surface of the membrane and suspended filler [21,22]. Compared to Proteobacteria, Bacteroidetes have a relatively lower abundance in the system but still represent the dominant species, especially in C1 and C2, with a relative abundance of 16.6% and 18.2%, respectively. Bacteroidetes are chemotrophic heterotrophic bacteria, capable of degrading large-molecule organic matter under anaerobic conditions [23]. Furthermore, by comparing the relative proportions of bacteria involved in carbon reduction-related metabolic pathways under optimal operating conditions between the HMBR and MBR, it is observed that the total proportion of functionally relevant bacteria in the MBR system constitutes merely 45.71% of that in the HMBR system. From these findings, it can be concluded that this spatial partitioning effectively enhances total active biomass and, compared to MBR, establishes longer and more diverse carbon degradation pathways.

**Figure 11:**
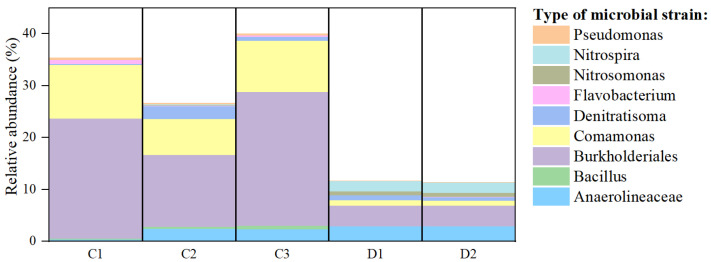
Diagram of the species composition of differential nitrification and denitrification metabolic pathways in the HMBR and MBR.

More notably, the removal rates of NH_4_^+^-N and TN in the HMBR reached 98.9–99.3% and 48.2–55.7%, respectively, significantly surpassing those of the MBR process (85.9% and 29.2%). This performance can be demonstrated through the species composition analysis of differential metabolic pathways shown in Figure 11, which indicates that the abundance of both nitrifying and denitrifying bacteria in the HMBR is significantly higher than that in the MBR. *Burkholderiales* occupied a significant portion of each sample’s microbial biomass, especially in sample C3. *Burkholderiales* play a significant role in the denitrification process. These bacteria not only utilize various organic carbon sources as electron donors to directly participate in denitrification reactions but also possess flocculation capabilities [24]. They can secrete EPS and enzymatic materials, promoting biofilm formation and providing a growth substrate for other denitrifying bacteria. Through these processes, electron transfer efficiency and reaction rates during denitrification have been enhanced, and the overall nitrogen removal pathway has been optimized. *Comamonas* can degrade complex nitrogen-containing organic pollutants [25]. Due to the differences in genetic composition, various *Comamonas* can degrade different pollutants, such as difficult-to-degrade aromatic compounds. In the composition of metabolic pathways, *Comamonas* constitute 10% of the total functional microbial composition in sample C1, 7% in sample C2, and 10% in sample C3, representing a proportion significantly higher than that of *Flavobacterium* in the corresponding samples. *Pseudomonas* presents good denitrification performance and is considered to be the strain with the most potential for synchronous nitrification and denitrification applications [26]. As shown in Figure 11, the relative abundance analysis of Pseudomonas based on metabolic pathway composition indicates that sample C2 exhibits the highest Pseudomonas content, followed by sample C1. Moreover, over 60% of *Pseudomonas* in C2 comprises uncultivated bacteria, indicating that aerobic denitrification bacteria on suspended fillers still have great potential [27]. *Anaerolineaceae* have been found to have the ability to degrade macromolecular organic compounds, such as glucose, providing more relatively small carbon sources for denitrification [28]. Therefore, increasing the abundance of *Anaerolineaceae* can further enhance microbial metabolic activity and improve denitrification capacity [29]. Within the relevant functional components, the relative abundance of *Anaerolineaceae* in C1, C2, and C3 is 0.40%, 2.47%, and 2.44% of the total microbial biomass, respectively. The amount of *Anaerolineaceae* in the suspended fillers is almost equal to that in the activated sludge mixture. Although the enrichment of denitrifying bacteria on the surface of suspended fillers is relatively low, 27.02% of all denitrifying microorganisms were still associated with the surface of the suspended fillers, indicating that they can promote the accumulation of denitrifying microorganisms and become a dominant microbial community and demonstrating that HMBRs have greater denitrification potential than MBRs.

**Figure 12:**
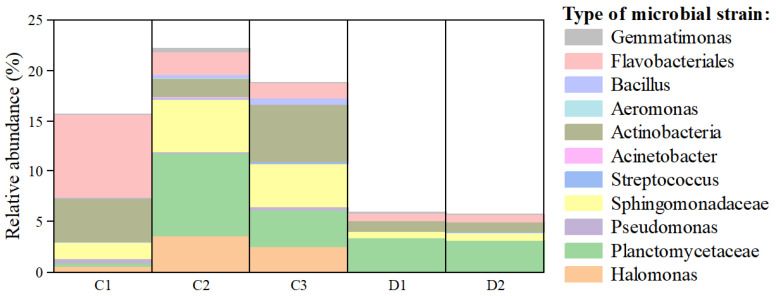
Diagram of the species composition of differential phosphorus removal metabolic pathways in the HMBR and MBR.

The biological phosphorus removal efficiency in the HMBR has been significantly enhanced—achieving total phosphorus removal rates of 78.3% to 83.3%, compared to merely 65% in the MBR—with its underlying mechanism rooted in the directed spatial enrichment of PAOs. The distribution pattern of PAOs is shown in Figure 12. From the perspective of metabolic pathway composition, the majority of phosphorus removal is attributed to metabolic pathways contributed by Planctomycetaceae, Flavobacteriales, Actinobacteria, and *Halomonas*. PAOs accounted for 15.09% of the total microbial functional composition in C1, 20.85% in C2, and 17.42% in C3. The sum of these bacteria in C1, C2, and C3 accounts for 25.98% of the total microbial population in the HMBR system, significantly higher than those in MBR. Among these, C2 exhibited the highest abundance of phosphorus-removing functional bacteria, with variations observed in the bacterial communities possessing phosphorus removal potential across different locations. *Flavobacteriales* show a preference for the membrane surface, *Planctomycetaceae* show a preference for suspended fillers’ surface sludge, and *Actinobacteria* are more suitable for survival in activated sludge mixtures. *Actinobacteria* were once considered to be the main genus of bacteria with phosphorus removal functions in biological phosphorus removal processes. In later work, Rusten et al. [30] found that γ-proteobacteria constituted 5% to 15% of the relevant metabolic pathways in the aerobic reactors of sewage treatment plants, which is consistent with the detection results presented in this study. In addition, *Actinobacteria* contribute significantly to the phosphorus removal function within activated sludge systems, where they have been identified as key PAOs in enhanced biological phosphorus removal processes [31]. However, Lee et al. [32] postulate that the composition ratio is not the only factor that determines the phosphorus removal function and that the phosphorus removal process is performed by *Actinobacteria* and *Flavobacteriales*, which are not in a dominant position. Therefore, although the proportions of *Actinobacteria* and *Flavobacteriales* in relevant metabolic pathways within this system are fairly low, their phosphorus removal effect cannot be ignored or disputed. The above findings indicate that the HMBR has greater potential for phosphorus removal than the MBR, which is closely associated with the higher proportion of phosphorus-removing functional microbial communities enriched within its suspended media.

**Figure 13:**
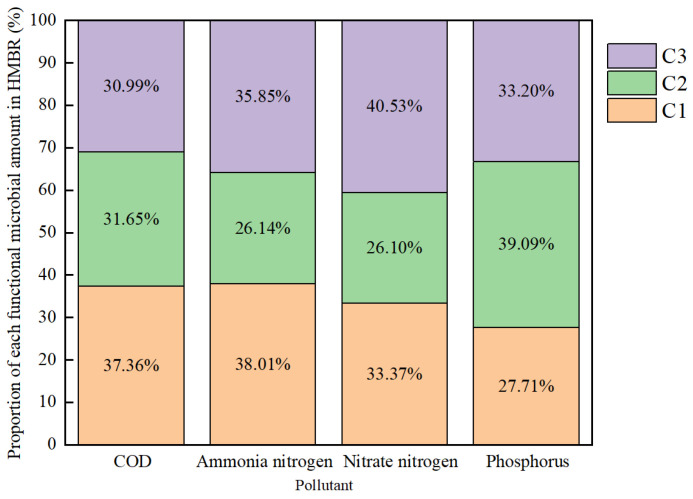
Prediction of the relative contribution of different microbial ecological niches to pollutant removal in the HMBR based on functional microbial abundance.

Based on the relative abundance of functional microorganisms across each ecological niche, Figure 13 estimates the respective contributions of suspended sludge (C3), carrier biofilm (C2), and membrane surface (C1) to pollutant removal. The carrier biofilm contributed approximately one-third of the total removal capacity for COD_Cr_ (31.65%), ammonia nitrogen (26.14%), and nitrate nitrogen (26.13%). It also bore the highest share of phosphorus removal across all zones—reaching 39.09% confirming the carrier biofilm as the primary hotspot for biological phosphorus removal. By contrast, in the conventional MBR system without carriers, the membrane surface bore nearly 50% of the estimated functional load. This comparison demonstrates that adding suspended media effectively shifts treatment loads away from the membrane surface, with carriers assuming the bulk of carbon, nitrogen, and particularly phosphorus removal functions. This functional shift reduces metabolic density on the membrane surface, thereby indirectly extending the filtration cycle of the HMBR and mitigating membrane fouling (Figure 2).

### 3.3. Implications for HMBR and MBR Technology

The findings of this study indicate that the superior performance of the HMBR stems not merely from increased biomass but fundamentally from alterations in the spatial structure and function of the microbial community. The introduction of suspended carriers effectively constructs a functionally partitioned microecosystem: the biofilm on the packing material provides a stable attachment habitat for aerobic autotrophic nitrifying bacteria and phosphorus-accumulating organisms, while suspended sludge flocs and membrane surfaces dominate the heterotrophic denitrification process. This physical spatial separation effectively coordinates the previously competing aerobic and anaerobic metabolic processes, enabling their synchronous and efficient operation. Consequently, the higher diversity, more uniform, and functionally specialized community structure maintained by the HMBR constitutes the direct microbiological basis for its capacity to achieve simultaneous, efficient carbon reduction, denitrification, and phosphorus removal.

## 4. Conclusions

Our study findings reveal distinct spatial partitioning within the HMBR microbial community, which is not randomly formed but closely linked to ecological functions. Suspended carriers serve as key habitats for nitrifying bacteria and phosphorus-accumulating bacteria; in comparison, denitrification predominantly occurs on suspended sludge and membrane module surfaces. This functional spatial architecture enables synergistic pollutant removal within the HMBR. While correlation analyses provide compelling evidence, subsequent studies are required to validate the actual metabolic activities of these spatially distinct communities. Our findings indicate that optimizing HMBR technology hinges upon refining distinct microbial ecosystems.

## Figures and Tables

**Table 1:** Operational parameters of the HMBR and MBR.

Index	Value
Effective volume	0.01 m^3^
Volume ratio of fillers	50.0%
Membrane area	1.0 m^2^
Flow rate	0.01·m^3^ s^−1^
Hydraulic retention time (HRT)	10 h
Sludge retention time (SRT)	10 days/30 days

**Table 2:** Characteristics of raw wastewater.

Index	Range
Temperature (°C)	20.1–28.5
pH	7.3–7.7
Dissolved oxygen (mg/L)	4.0
TSS (mg/L)	27.8–70.0
COD_Cr_ (mg/L)	260.4–320.2
BOD_5_	12.7–40.1
NH_4_^+^-N (mg/L)	38.7–87.5
TN (mg/L)	58.1–104.3
TP (mg/L)	4.7–6.3

Abbreviations: TSS, total suspended solids; COD_Cr_, Chemical Oxygen Demand with Potassium Dichromate; BOD_5_, Biochemical Oxygen Demand (5 days); NH_4_^+^-N, ammonia nitrogen; TN, total nitrogen; TP, total phosphorus.

**Table 3:** Polymerase chain reaction (PCR) setup.

Reaction Component	First PCR Amplification	Second PCR Amplification
5× Buffer	10 μL	8 μL
dNTP (10 mM)	1 μL	1 μL
Phusion super-fidelity DNA polymerase	1 U	0.8 U
Primers (10 μM)	1 μL each (inner primers)	1 μL each (outer primers)
Template	5–50 ng	5 μL
ddH_2_O	Supplement to 50 μL	Supplement to 40 μL

**Table 4:** PCR amplification conditions.

First PCR Amplification				Second PCR Amplification			
Reaction Stage		Temperature (°C)	Time	Reaction Stage		Temperature (°C)	Time
Amplification (22 cycles)	Pre-denaturation	94	2 min	Amplification (8 cycles)	Pre-denaturation	94	2 min
	Denaturation	94	30 sec		Denaturation	94	30 sec
	Annealing	55	30 sec		Annealing	56	30 sec
	Extension	72	30 sec		Extension	72	30 sec
Final extension	Final extension	72	5 min	Final extension	Final extension	72	5 min
Preserve	Preserve	10	/	Preserve	Preserve	10	/

**Table 5:** Organic removal performed by the MBR and HMBR.

Project		COD_cr_	NH_4_^+^-N	TN	TP
MBR (HRT = 10 h, SRT = 10 days)	Average discharge (mg/L)	32.6	5.7	52.7	2.1
	Average removal rate (%)	87.5	85.9	29.2	65
HMBR (HRT = 10 h, SRT = 10 days)	Average discharge (mg/L)	13.2	0.6	32.8	1.3
	Average removal rate (%)	94.6	98.9	48.2	78.3
HMBR (HRT = 10 h, SRT = 30 days)	Average discharge (mg/L)	9.8	0.4	28.8	1.0
	Average removal rate (%)	96.1	99.3	55.7	83.3

**Table 6:** Alpha diversity index of different samples.

Sample	Coverage	Sobs	Chao	Ace	Shannon	Simpson	PD-Whole-Tree
C1	0.995	1174	1532.705	1536.840	4.691	0.071	111.947
C2	0.994	1503	2001.080	1985.090	5.226	0.015	135.630
C3	0.993	1661	2189.448	2159.254	4.956	0.028	144.861

## Data Availability

All relevant data are included in the paper.
